# Immune Thrombocytopenia Secondary to COVID-19 in a Vitamin B12-Deficient Patient: A Diagnostic Dilemma and Therapeutic Challenge

**DOI:** 10.7759/cureus.40199

**Published:** 2023-06-09

**Authors:** Mohanty Bijaya, Zeya Ansari, Binu Koshy, Ashok Sunder

**Affiliations:** 1 Internal Medicine, Tata Main Hospital, Jamshedpur, IND; 2 Internal Medicine, Manipal Tata Medical College, Jamshedpur, IND

**Keywords:** covid 19, azathioprine, immunoglobulin, methyl prednisolone, thrombocytopenia, treatment, itp, cobalamin, vitamin b12

## Abstract

Immune thrombocytopenia (ITP) caused by infectious and non-infectious conditions has been reported in coronavirus disease 2019 (COVID-19) patients too. Here we present a 64-year-old male patient with post-COVID-19 pneumonia who presented with a gastrointestinal bleed and was found to have severe isolated thrombocytopenia (22,000/cumm) diagnosed as ITP with extensive investigations. He was treated with pulse steroid therapy and later was also given intravenous immunoglobin in view of poor response. The addition of eltrombopag also resulted in a sub-optimal response. He was also having low vitamin B12, and his bone marrow also supported the megaloblastic picture. Hence, injectable cobalamin was added to the regimen, which resulted in a sustained rise in platelet count that reached 78,000/cumm, and the patient got discharged. This shows the possible hindrance to treatment response by concomitant B12 deficiency. Vitamin B12 deficiency is not an uncommon entity and should be tested in those who show no or slow response to thrombocytopenia.

## Introduction

Immune thrombocytopenia (ITP) also known as idiopathic thrombocytopenic purpura, can be primary or secondary. Primary ITP is characterized by isolated thrombocytopenia (peripheral blood platelet < 100x10⁹/L) in the absence of other causes or disorders that may be associated with it [1].

All other ITP except primary is known as secondary thrombocytopenia. It is found to be associated with several viral infections caused by HIV, hepatitis C virus, cytomegalovirus (CMV), Epstein-Barr virus, *Helicobacter pylori*, and many others with the latest addition of severe acute respiratory syndrome coronavirus 2 (SARS CoV-2).

While most patients who become infected with coronavirus disease 2019 (COVID-19) recover without complications, some patients develop sequelae. One such complication of COVID-19 is ITP [2]. ITP is caused by autoantibodies against platelet antigens, resulting in thrombocytopenia. Acute ITP appears abruptly, often one to two weeks after a self-limited viral illness. The illness appears to trigger the development of autoantibodies through uncertain mechanisms. The incidence of thrombocytopenia in COVID-19 infections is not well established as there is variability in different studies. The incidence of primary ITP is 3.3 per 100,000 adults per year, with a prevalence of 9.5 per 100,000 adults [3].

Mild thrombocytopenia has been noted in up to 33% of these patients but is more common and severe in patients with severe COVID-19 disease (57.7%) compared with nonsevere disease (31.6%) [4]. We present a case of a 64-year-old male who presented with complaints of black stool for two days. He was found to have severe thrombocytopenia (platelet count 22,000/cumm). He was a known diabetic, had COVID-19 pneumonia, and was discharged a week ago. He was found to have a very low level of serum cobalamin and further investigations revealed megaloblastic anemia with thrombocytopenia. He was treated with pulse steroid therapy, intravenous immunoglobulin (IVIg), eltrombopag, azathioprine, and vitamin B12. He improved well with judicious management and was discharged.

## Case presentation

A 64-year-old male got admitted with complaints of black stool for two days. He was a known diabetic patient and was on oral antidiabetic agents. There was no history of non-steroidal anti-inflammatory drugs (NSAIDs) or any alternative drug abuse. There was no fever, vomiting, or pain abdomen. He had suffered from moderate COVID-19 pneumonia and got discharged seven days prior to the current admission. He was treated with IV methylprednisolone 40 mg twice a day, IV remdesivir 100 mg once a day, injection insulin (rapid-acting) as per sliding scale, and low-molecular-weight heparin (LMWH) 40 mg subcutaneously once a day for five days during active COVID-19 infection. He was also prescribed oral vitamin C 500 mg and zinc acetate 50 mg once daily as per the protocols laid by the World Health Organization (WHO) and the Ministry of Health & Family Welfare, Government of India. On discharge from the COVID-19 isolation ward, he was prescribed apixaban 5 mg once daily, prednisolone 40 mg once a day, oral antidiabetics, vitamin C, and zinc.

In this admission, he was conscious, alert, and afebrile. There was no pallor, icterus, clubbing, lymphadenopathy, or pedal edema. His pulse was 100/minute and blood pressure was 130/80 mmHg. Systemic examination did not reveal any abnormality. There was no ecchymosis or purpura.

Routine blood investigation revealed severe thrombocytopenia (platelet count 22,000/cumm). Hemoglobin, total leucocyte count, and reticulocyte count were within normal limits. On his discharge for COVID-19, his platelet count was 1,68.000/cumm. The details of the hemogram of current admission and previous discharge after COVID-19 pneumonia have been shown in Table 1.

**Table 1:** Comparision of hemogram of previous discharge after COVID-19 and current admission COVID-19: coronavirus disease 2019

Hemogram parameter	On the day of discharge after COVID-19 infection	On the first day of current admission
Hemoglobin	12.4 gm/dl	12 gm/dl
Total leucocyte count	6900/cumm	6200/cumm
Neutrophil	81%	79%
Lymphocyte	13%	18%
Monocyte	5%	2%
Eosinophil	1%	1%
Basophil	0%	0%
Mean platelet volume	11.50 fl	12.3 fl
Mean corpuscular volume	85.7 fl	86 fl
Platelet count	168,000/cumm	22,000/cumm

His coagulation profile including prothrombin time (PT), activated partial thromboplastin time (aPTT), d-dimer, and serum fibrinogen level was within normal range. He also had normal liver and renal function tests. Besides these, tests for malaria and dengue were also negative but vitamin B12 was low (86 pg/ml). Chest X-ray showed clear lung fields. Ultrasound of the abdomen and pelvis revealed prostatomegaly. There was no hepatosplenomegaly. He was treated with pantoprazole infusion at the rate of 8 mg/hour, one unit of single donor platelet along with IV methylprednisolone (1 gm) as pulse for three days followed by oral steroid 60 mg once daily. After three days of pulse steroid therapy, platelet count improved to 58,000/cumm. Upper gastrointestinal endoscopy was done, which revealed antral gastritis and oesophageal candidiasis. Fluconazole 150 mg and vitamin B12 1500 mg oral supplementation once daily were added to the treatment regimen. On the fifth day of admission, the platelet counts again started showing a downside trend (29,000/cumm).

Bone marrow aspiration and trephine biopsy were advised to rule out ITP, hematological malignancy, and myelodysplasia. On the seventh day, there was a further fall in his platelet count (27,000/cumm). Antiplatelet factor 4 (Anti PF-4) antibody was given in view of recent heparin use and the probability of heparin-induced thrombocytopenia.

**Figure 1:**
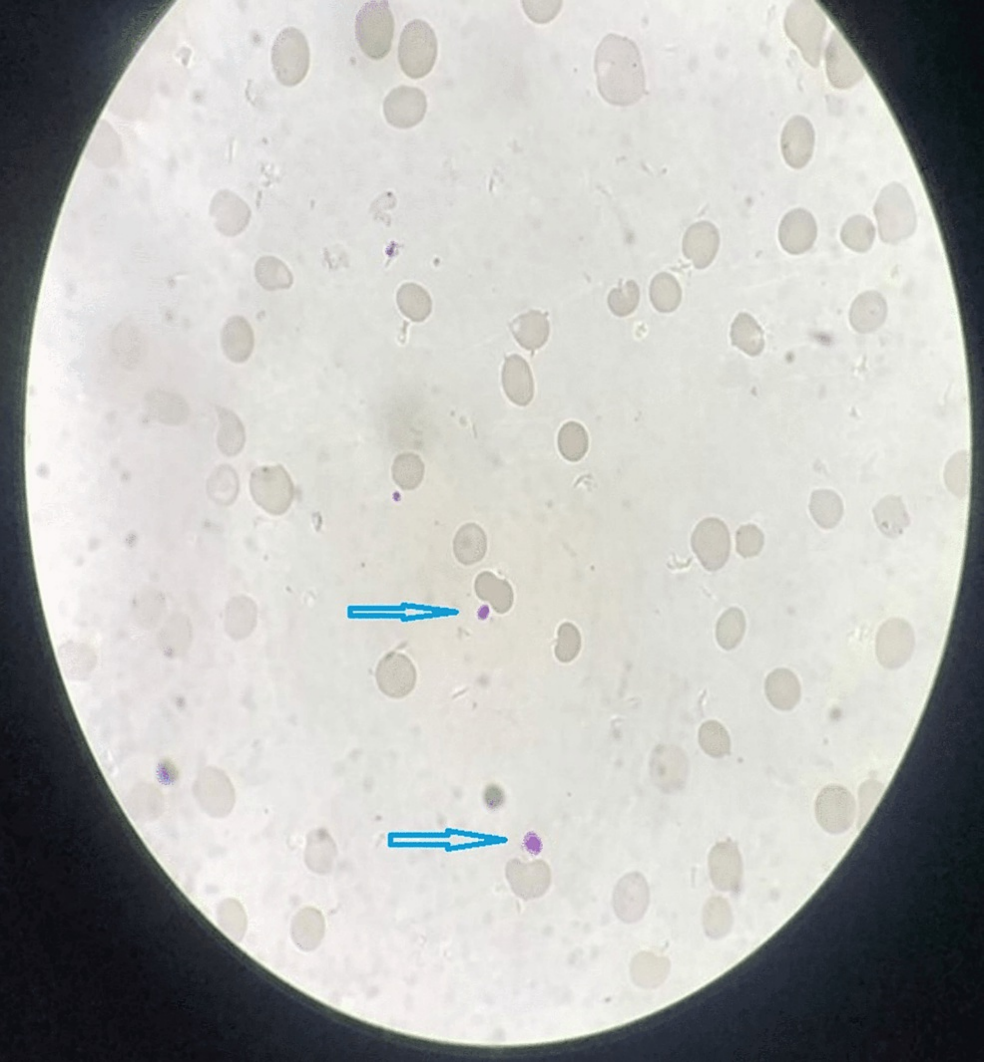
Peripheral blood smear (1000x oil immersion, Leishman stain) showed few large platelets (blue arrows) without atypical cells and parasites

**Figure 2:**
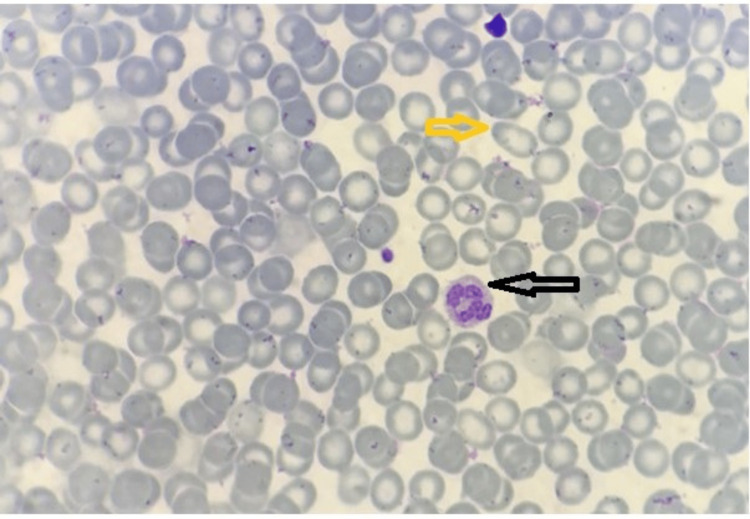
Peripheral blood smear (1000x oil immersion, Leishman stain) RBC: normocytic, few microcytes, hypochromia, elliptocyte (yellow arrow); WBC: hypersegmented neutrophils (black arrow); platelets: reduced

Peripheral blood smear (Figures 1-2) showed a few large platelets without atypical cells and parasites, and hypersegmented neutrophils, elliptocytes, and reduced platelets.

**Figure 3:**
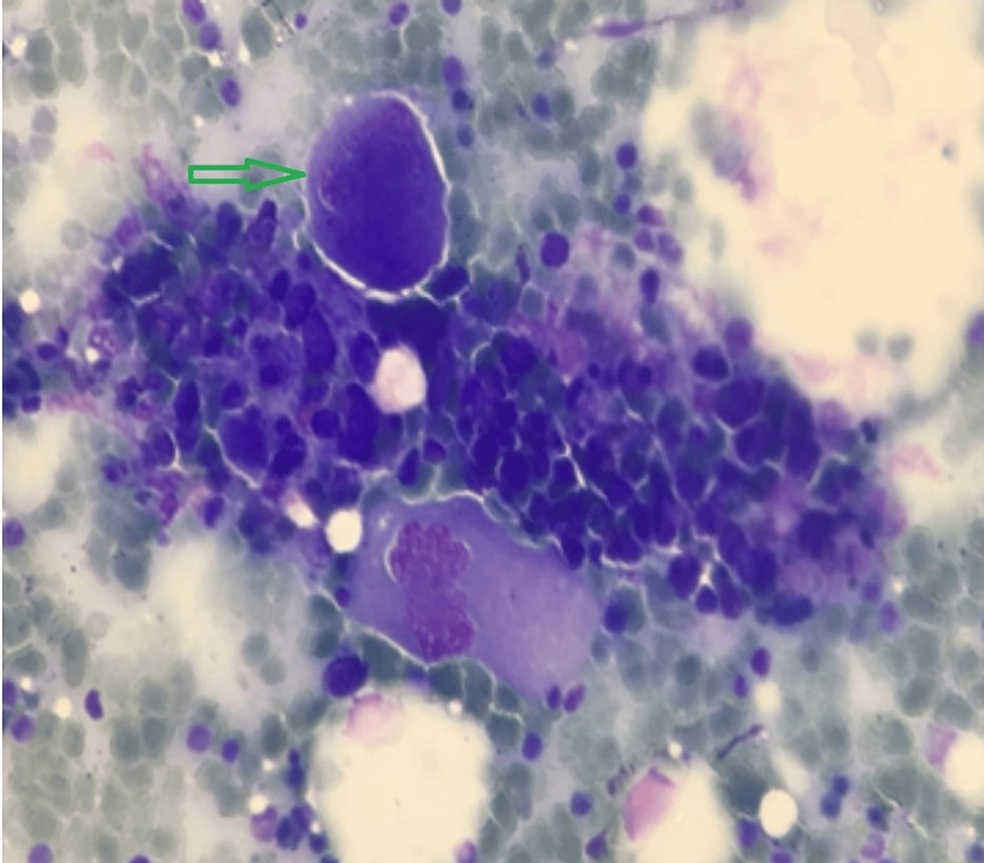
Bone marrow smear (1000x oil immersion, Leishman stain) showed normocellular to hypercellular particles, hypoglobated megakaryocyte (green arrow), and erythroid colonies

Bone marrow examination (Figure 3) showed thrombocytopenia with trilineage differentiation, dimorphic erythropoiesis with the presence of few megaloblasts, elliptocytes, and hypersegmented neutrophils without atypical cells, schistocytes, and parasites suggestive of megaloblastosis and thrombocytopenia.

**Figure 4:**
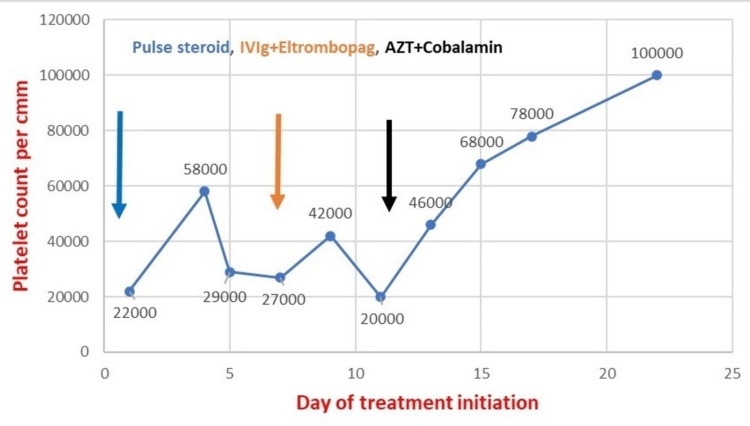
Line diagram showing response to therapy IVig: intravenous immunoglobulin; AZT: azathioprine cmm=cumm=cubic millimeter

With the possibility of ITP and falling platelet counts (24,000/cumm), IVIg 1 gm/kg body weight (70 gm) was given over four to six hours for two consecutive days. Eltrombopag 25 mg one tablet orally was started. After two days of IVIg, platelet count increased to 42,000/cumm. His CMV polymerase chain reaction (PCR) qualitative and chromosomal analysis for hematological malignancy was also done. After four days of IVIg, platelet count decreased to 20,000/cumm, and hence azathioprine 50 mg one tablet daily was added and oral cobalamin was changed to IV B1, B6, and B12 combination. Anti PF-4 antibody was not found, CMV PCR was negative, and chromosomal analysis was reported as normal. After two days of azathioprine, platelet count increased to 46,000cumm. After four days of azathioprine and B complex, platelet count started increasing gradually to 68,000/cumm, and then 78,000/cumm. The response to therapy is shown in Figure 4.

The patient was discharged on medications. He was followed up in the outpatient department initially on a weekly basis for four weeks, and then later every month for six months. At present, he is doing well and his platelet count is more than 100,000/cumm.

## Discussion

ITP has been reported in COVID-19 patients and the incidence is different in various studies from different parts of the world. This can happen during active COVID-19 infection or may ensue after recovery. Since the very first case of COVID-19-associated ITP, reported by Zulfiqar et al. [5], several cases have been reported from all over the world. Our patient was diagnosed with ITP associated with cobalamin deficiency after excluding other possible causes of isolated thrombocytopenia and was on treatment as per indication. The association of ITP with cobalamin deficiency is not mentioned in previous studies, though there is a definite impact of cobalamin deficiency on hematological parameters like macrocytosis, ovalocytosis, pancytopenia, or bicytopenia with thrombocytopenia; however, seeing isolated thrombocytopenia is very less likely. If there is a coexistence of ITP and cobalamin deficiency, the response to therapy may not be up to the expected level and pose a problem of no response to therapy and a longer hospital stay. Although the mechanism behind cytopenia in cobalamin deficiency is defective DNA synthesis, which is a well-known factor, thrombocytopenia in COVID-19 has been postulated to be due to various mechanisms. These could be bone marrow infection by a coronavirus, formation of micro-thrombi in the lungs, or an immune complex that may either decrease thrombopoiesis or increase platelet destruction leading to thrombocytopenia [6,7].

International Working Group (IWG) defines a refractory ITP as one that does not respond to or relapses after splenectomy and that requires treatment to reduce the risk of clinically significant bleeding. Our patient had a fluctuating trend of thrombocytopenia with no desired level of therapeutic response to combined IV steroid and IVIg. Azathioprine, eltrombopag, and parenteral cobalamin were added to his treatment regime, following which he started showing the expected rise in platelet count. Although we are not sure which one of the cocktail therapy of the three medications, the thrombocytopenia responded to, the role of cobalamin cannot be denied. In contrast to our case, most of the other studies on ITP in COVID-19 have mentioned initial good response in thrombocytopenia to either IV steroid or a combination of it with Ig [8,9].

A review of the literature shows cases where cobalamin deficiency may mimic thrombotic microangiopathy, thrombotic thrombocytopenic purpura, and even acute leukemia; however, its association with isolated thrombocytopenia remains a matter of research [10,11]. Such association has also been refuted in a review article by De Loughery et al. [12]. There are cases of isolated thrombocytopenia in children reported from Turkey but with extremely rare (0.9%) incidence [13,14].

A study on dengue fever with cobalamin deficiency showed significant improvement in hematological parameters including thrombocytopenia after administering vitamin B12 to the patients [15]. Ayesh and Alawneh have reported two incidents of chronic ITP in cases of vulvovaginal candidiasis in contrast to our case which had oesophageal candidiasis and acute ITP [16]. An article on *Candida* and platelet interaction has shown that *Candida*-derived secretory products did not affect platelet activity neither stimulatory nor inhibitory [17]. 

This report throws some light on the reasons behind some of the cases of ITP being either no or slow responders to the conventional treatment regimen in the background of hidden or unveiled cobalamin deficiency

## Conclusions

Patients with secondary ITP many a times may have associated conditions like vitamin B12 deficiency that may add to thrombocytopenia. Until and unless we address such issues concomitantly, we may not get the desired level of response to therapy. Hence, if the desired level of response with standard therapy is not achieved, other associated conditions need to be identified and treated to get the optimum result.
